# Dengue and chikungunya viruses among febrile travelers entering Iran (2015-2021): Evidence of multiple introductions from different countries

**DOI:** 10.1016/j.ijregi.2025.100593

**Published:** 2025-02-01

**Authors:** Tahmineh Jalali, Mohammad Hasan Pouriayevali, Marzyie Sajadi, Sepideh Gerdooei, Tahereh Mohammadi, Mahsa Tavakoli, Sahar Khakifirouz, Laya Farhan Asadi, Mohammad Sadegh Shamas Nosrati, Sana Eybpoosh, Mostafa Salehi-Vaziri

**Affiliations:** 1 Department of Arboviruses and Viral Hemorrhagic Fevers (National Reference Laboratory), Pasteur Institute of Iran, Tehran, Iran; 2 Department of Neurosciences, Rehabilitation, Ophthalmology, Genetics, Maternal and Child Health, University of Genoa, Genoa, Italy; 3 U.O.C. Genetica Medica, IRCCS Istituto Giannina Gaslini, Genoa, Italy; 4 Department of Epidemiology and Biostatistics, Research Centre for Emerging and Reemerging Infectious Diseases, Pasteur Institute of Iran, Tehran, Iran

**Keywords:** Dengue, Chikungunya, Zika, Travel related disease, Multiplex real-time RT-PCR, Diagnosis

## Abstract

**Objectives:**

Recent outbreaks of dengue, chikungunya, and Zika have underscored the growing threat to public health. This study retrospectively describes the presence of these viruses among febrile travelers entering Iran between 2015 and 2021. Although Iran had no local transmission until 2024, several factors increased the risk of outbreaks, including the presence of mosquito vectors, favorable climate, and inadequate traveler control measures. Climate change and increased humidity have further increased this risk.

**Methods:**

A multiplex real-time reverse transcription-polymerase chain reaction assay was used to screen 245 serum samples from febrile travelers, identifying dengue and chikungunya viruses in 13.9% of the travelers.

**Results:**

Dengue was more prevalent (9%) than chikungunya (4.9%), but no Zika infections were detected. Genetic analysis identified three dengue virus (DENV) serotypes (DENV-1, DENV-2, and DENV-3) and an Asian genotype of chikungunya virus-like strains from the 2017 outbreak in Pakistan. Pakistan was the primary source of chikungunya (100%) and a significant source of dengue (37%). Travelers from Southeast Asia also contributed to the remaining dengue cases.

**Conclusions:**

This study highlights the risk of importing dengue and chikungunya viruses into Iran, particularly through the land border with Pakistan. The endemicity of these viruses in neighboring countries and high cross-border movement significantly increase the risk of outbreaks in Iran.

## Highlights

• The Iran-Pakistan border is a major entry point for dengue and chikungunya viruses.
• Higher dengue and chikungunya in men aged 21-40 due to travel and social activity.
• Disease referrals significantly dropped in 2020 due to the pandemic.
• Positive cases rise during rainy, hot months, needing intensified control measures.

## Introduction

Dengue, Chikungunya, and Zika viruses are the major mosquito-borne arboviruses transmitted by *Aedes* mosquitoes. Recent large-scale outbreaks have highlighted these viruses as a serious threat to public health. *Aedes aegypti* and *Aedes albopictus* mosquitoes are the primary vectors for these viruses, and the geographic distributions of Dengue, Chikungunya, and Zika are linked to the prevalence of these vectors [1]. Several factors significantly contribute to the spread of these vectors, and consequently, the arboviruses, Dengue, Chikungunya, and Zika. These factors include global warming, globalization, significant population growth, increased travel, and international trade [[2], [3], [4]].

Although no local transmission of these viruses was reported in Iran until 2024, their potential for introduction and the high risk of outbreaks, potentially leading to endemicity, pose a serious threat. Several factors further amplify this threat, including the identification of *Aedes albopictus* larvae and adult mosquitoes in Sistan, Baluchestan, Gilan, and Mazandaran provinces; detection of *Aedes aegypti* larvae and adult mosquitoes in Sistan, Baluchestan, Hormozgan, and Bushehr provinces [1]; favorable climatic conditions for vector establishment in the region; insufficient control measures for inbound travelers from endemic countries; global risk of climate change that influences vector globalization, density, bite rate, and virus fitness; and international tourism and travel increasing the risk of virus introduction by infected travelers and vector spread. The potential introduction of Dengue, Chikungunya, and Zika viruses into Iran raises significant concerns owing to the country's vulnerability to these diseases. The presence of competent vectors, favorable climatic conditions, and inadequate vector control measures create a substantial risk of outbreaks and eventual endemicity. In addition, the lack of public awareness and preparedness further exacerbates this threat.

In this study, the presence of Dengue, Chikungunya, and Zika viruses was investigated retrospectively among febrile travelers entering Iran from 2015 to 2021 using biobank's sample of arboviruses and viral hemorrhagic fevers laboratory (National ref lab), Pasteur Institute of Iran, and in house multiplex real-time reverse transcription-polymerase chain reaction (RT-PCR) assay. Viral load was assessed, and positive samples with sufficient load were sequenced for serotype/genotype identification and phylogenetic analysis. In this study, we describe founded positive cases in terms of population characteristics, visiting areas, time periods, and virus genotypes.

## Methods

### Sample and information collection

Following national guidelines for arboviral diseases and viral hemorrhagic fevers surveillance, serum samples were collected from acute febrile patients with two or more of the following symptoms: severe headache, retro-orbital pain (pain behind the eyes), gastrointestinal symptoms such as nausea and vomiting, myalgia, weakness, fatigue, arthralgia/severe bone pain, a maculopapular or morbilliform skin rash, hemorrhagic symptoms from various sites such as nosebleeds, gum bleeding, leukopenia (≤5,000 cells/mm^3^), thrombocytopenia (≤150,000 cells/mm^3^), a positive tourniquet test, and increased hematocrit, along with at least one of the following: positive serology (positive IgM and/or IgG in a single sample) or a positive epidemiological history (residing or traveling in the past 2 weeks to endemic areas, regions with local transmission of the disease, areas infested with *Aedes* mosquitoes, or residing in a high-risk area). The samples were collected prospectively from patients seeking care at health centers and hospitals across various regions of Iran as part of the routine clinical surveillance for arboviral infections. The serum samples for this study included febrile patients who were referred to the National Reference Laboratory due to clinical suspicion of arboviral infections between January 2015 and January 2021 and who had fever and travel history.

A detailed protocol was followed for sample collection. Approximately 10 ml of blood was drawn from each patient, and the serum was separated, divided into three cryotubes, carefully sealed with parafilm, and labeled with patient information. The samples were then transported under cold-chain conditions to the Arboviruses and Viral Hemorrhagic Fevers Department at the Pasteur Institute of Iran. Each sample was accompanied by a complete information form containing demographic and clinical data, including travel history and febrile symptoms. The samples were stored at −70°C until further analysis.

### Ethical consideration

The study protocol was approved by the research and ethics committee of the Pasteur Institute of Iran (ethical code: IR. PII. REC.1400.030). As an established ethical practice, consent was obtained from all suspected cases to provide serum samples and information for the national surveillance program. The samples were anonymized for analysis.

### RNA extraction

RNA was extracted from serum samples using the commercial QIAamp® Viral RNA Mini Kit (Cat No: 52906) from QIAGEN.

### Multiplex assay design

A real-time RT-PCR molecular assay using the TaqMan method with primers and probes obtained from previous studies [[5], [6], [7]] (Supplementary Table 1) was designed and validated to detect acute infections caused by dengue, chikungunya, and Zika viruses. The in-house assay showed 100% specificity and sensitivity (data not shown). The reaction mix was prepared using the 4X CAPITAL™ 1-Step qRT-PCR Probe Master Mix (Cat No: BR0502001) from BiotechRabbit, and the reaction components were as follows: 5 µl CAPITAL qRT-PCR Probe Mix 4X, 1 µl RTase with RNase inhibitor 20X, 0.4 µl of each primer, and 0.2 µl of each probe with 10 µM concentration and 2 µl template in 20 µl total reaction volume. Followed by the thermal cycler program, 10 minutes at 50°C for Revers Transcription, 3 minutes at 95°C for initial denaturation, and 45 cycles of 10 seconds at 95°C and 30 seconds at 60°C with acquiring on the yellow, green, red, and orange channel.

### Sequencing

For partial genome sequencing of the detected viruses, positive samples were amplified using the 1-Step RT-PCR Master Mix (Cat No: BR0400102) from BiotechRabbit and specific primers [[8], [9], [10]] (Supplementary Table 2). For each 25 µl reaction mix, 12.5 µl onestep mix 2x, 1 µl of each primer with 5 µM concentration, 1.25 µl enzyme RT-RI blend 20X, and 2.5 µl template were mixed. The thermal cycling program for amplification consists of the following steps: 20 minutes at 50°C for RT, 2 minutes at 95°C for initial denaturation, and 40 cycles of 10 seconds at 95°C, 10 seconds at 61/62°C (dengue/chikungunya), and 1 minute at 72°C followed by 5 minutes at 72°C as a final extension. Sequencing was performed using the ABI 3500 Genetic Analyzer. The CLC Main Workbench software was used to evaluate the sequencing results. The sequence alignment and phylogenetic reconstruction utilized the “build” function within ETE3, implemented on GenomeNet (https://www.genome.jp/).

The presence of dengue and chikungunya viruses among febrile travelers entering Iran was analyzed using SPSS software.

## Results

Samples from individuals with a history of travel during the study period were selected from the serum bank of the Arboviruses and Viral Hemorrhagic Fevers Department (National Ref Lab), Pasteur Institute, Iran. A total of 245 samples were analyzed, all tested for the presence of dengue, chikungunya, and Zika virus genomes. The sex distribution of the sample was 56.7% male (139 participants) and 43.3% female (106 participants). The nationalities of the patients were as follows: 74.2% (182 patients) Iranian, 5.6% (16 patients) Pakistani, 4.2% (six patients) Afghan, 0.4% (one patient) Iraqi, 0.4% (one patient) Thai, and 15.1% (37 patients) of unknown nationalities (all residing in Iran during sampling). In total, 13.9% of the patients had a positive real-time RT-PCR test for the studied viruses (dengue, chikungunya, and Zika). Of these, 9% (22 patients) were dengue virus-positive and 4.9% (12 patients) were chikungunya virus-positive. No cases of Zika virus infection were detected.

Among the positive cases, men seemed to bear the brunt of the illness slightly more often, with 58.9% (20 cases) compared with 41.1% (14 cases) in women (not significant). This trend was observed for both dengue and chikungunya infections. Regarding dengue, 59.1% (13 patients) were male, whereas 40.9% (nine patients) were female. Similarly, chikungunya showed a distribution of 58.3% (seven cases) male and 41.7% (five cases) female (not significant).

The average age of patients with both diseases was 33 years, with a range spanning from a young age of 1 year to a seasoned age of 71 years. Taking a closer look at the age distribution of positive cases, we observed that dengue struck most frequently in the 21-40 age group, accounting for 59.1% (13 patients) of the patients. This was followed by the >41 age group with 27.3% (six patients), and lastly, the <20 age group with the least number of cases at 13.6% (three patients). Chikungunya followed a similar pattern, with the highest number of positive cases (66.7%, eight patients) falling within the 21-40 age range. The >41 age group comprised 33.3% (four patients) of the chikungunya cases (Supplementary Figure 1).

**Figure 1:**
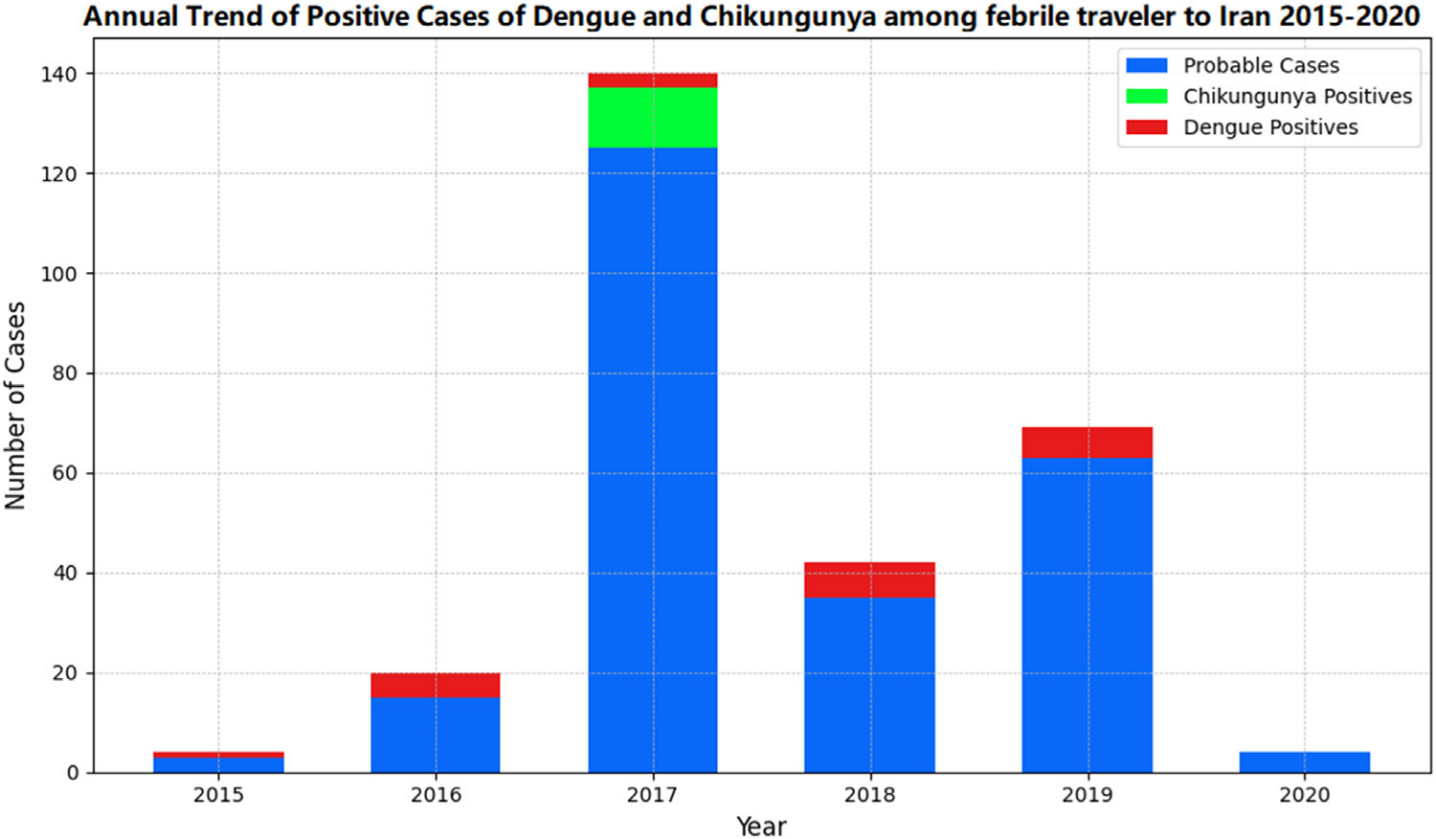
(a) Positive cases of dengue and chikungunya unfolded year by year from 2015 to 2021. (b) Monthly occurrence of positive cases of dengue and chikungunya from 2015 to 2021.

A more detailed picture of how these positive cases unfolded year by year and the seasonal presence of the disease is shown in Figure 1a and b.

**Figure 2:**
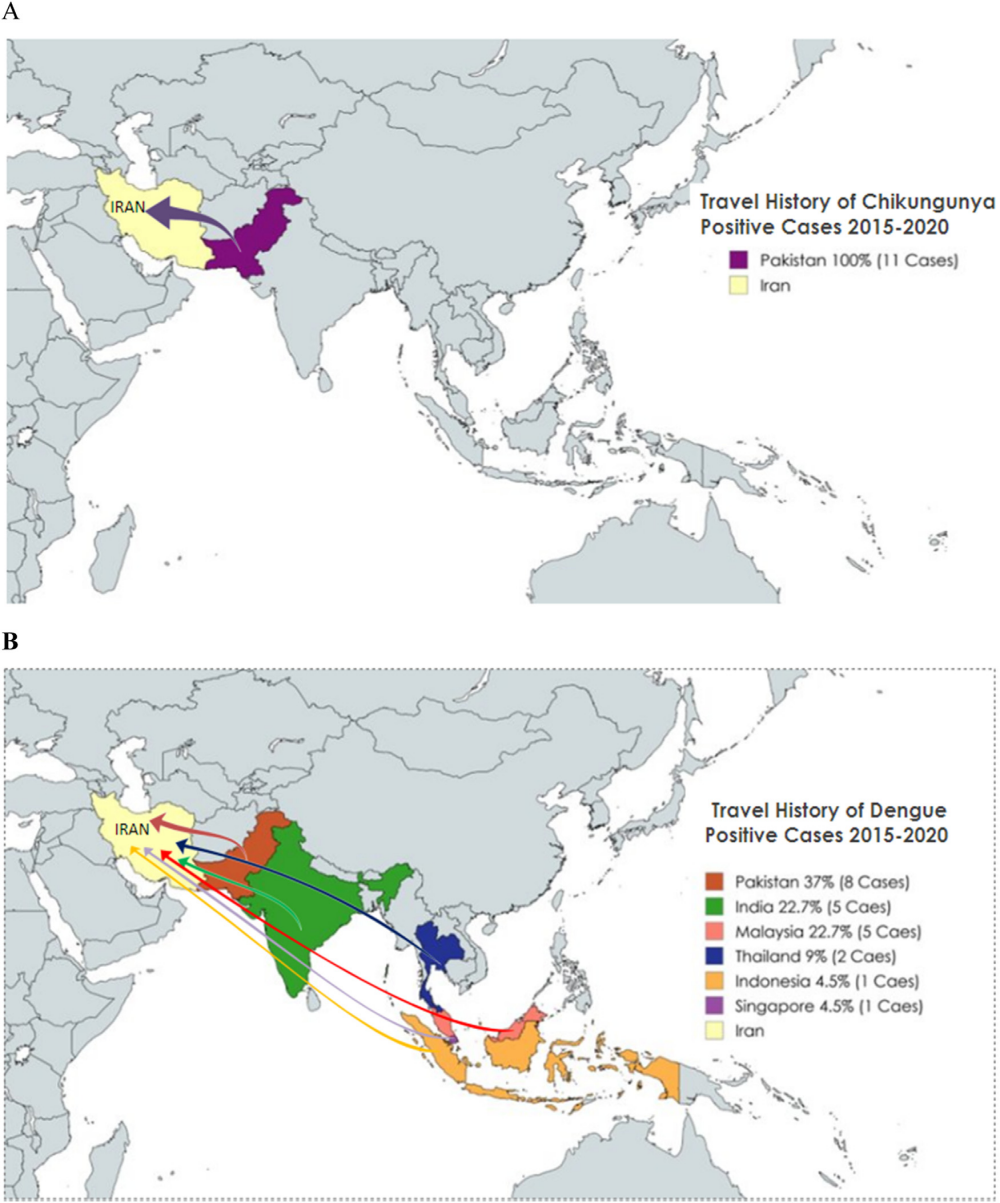
Imported cases of chikungunya (a) and dengue (b) to Iran from 2015 to 2021.

In this study, 13.9% of patients tested positive for the viruses under investigation using real-time RT-PCR. Among these, 9% (22 patients) tested positive for dengue virus and 4.9% (12 patients) tested positive for chikungunya virus. No cases of Zika virus infection were detected. All chikungunya-positive cases (12 CHIKV-positive cases) originated in Pakistan. Moreover, Pakistan accounted for 37% (eight cases) of the dengue-positive cases. The remaining dengue-positive cases were traced back to Malaysia and India, each contributing 22.7% (five cases). Thailand accounted for 9% (two cases), whereas Indonesia and Singapore accounted for 4.5% (one case) (Figure 2a and b). Analysis of transport modes revealed that land transport between Iran and neighboring countries accounted for 59% (20 cases) of the positive cases, whereas air travel accounted for 41% (14 cases). Phylogenetic analysis of the dengue virus serotypes revealed three distinct serotypes: 1, 2, and 3. The specific distribution of these serotypes by country of origin, year, and genotype is listed in Table 1. Lineage analysis was performed using the Genome Detective Platform (https://dengue-lineages.org/).

**Table 1:** Distribution of dengue-positive cases serotypes and genotypes by country of origin and year.

Accession number	Virus name	Den serotype	Den genotype	Country of origin	Year
EPI_ISL_19357700	hDenV3/Imported to Iran/Pakistan-6669/2018	Den 3	3III-B.1	Pakistan	2018
EPI_ISL_19357701	hDenV3/Imported to Iran/Pakistan-6793/2019	Den 3	3III-B.1	Pakistan	2019
EPI_ISL_19357702	hDenV1/Imported to Iran/Pakistan-6835/2019	Den 1	1III-A	Pakistan	2019
EPI_ISL_19357703	hDenV2/Imported to Iran/Pakistan-7384/2019	Den 2	2II-F.1	Pakistan	2019
EPI_ISL_19357704	hDenV2/Imported to Iran/Pakistan-7385/2019	Den 2	2II-F.1	Pakistan	2019
EPI_ISL_19357710	hDenV2/Imported to Iran/India-4679/2015	Den 2	2II-A.2.1	India	2015
EPI_ISL_19357711	hDenV1/Imported to Iran/India-5029/2016	Den 1	1I-H.1	India	2016
EPI_ISL_19357712	hDenV3/Imported to Iran/India-5062/2016	Den 3	3III-B	India	2016
EPI_ISL_19357713	hDenV1/Imported to Iran/India-7310/2019	Den 3	3III-B.3	India	2018
EPI_ISL_19357714	hDenV3/Imported to Iran/India-6617/2018	Den 1	1III-A	India	2019
EPI_ISL_19357709	hDenV1/Imported to Iran/Indonesia-4959/2016	Den 1	1I-K.1	Indonesia	2016
EPI_ISL_19357705	hDenV2/Imported to Iran/Malaysia-4996/2016	Den 2	2II-F	Malaysia	2016
EPI_ISL_19357706	hDenV3/Imported to Iran/Malaysia-5159/2017	Den 3	3I-A.1	Malaysia	2017
EPI_ISL_19357707	hDenV3/Imported to Iran/Malaysia-5270/2017	Den 3	3I-A	Malaysia	2017
EPI_ISL_19357708	hDenV2/Imported to Iran/Malaysia-6218/2018	Den 2	2II-F.1	Malaysia	2018
EPI_ISL_19357697	hDenV1/Imported to Iran/Thailand-6169/2018	Den 1	1I-K.1	Thailand	2018
EPI_ISL_19357698	hDenV1/Imported to Iran/Thailand-6514/2018	Den 1	1I-H.1	Thailand	2018
EPI_ISL_19357699	hDenV1/Imported to Iran/Singapore-4866/2016	Den 1	1I-K.1	Singapore	2016

**Figure 3:**
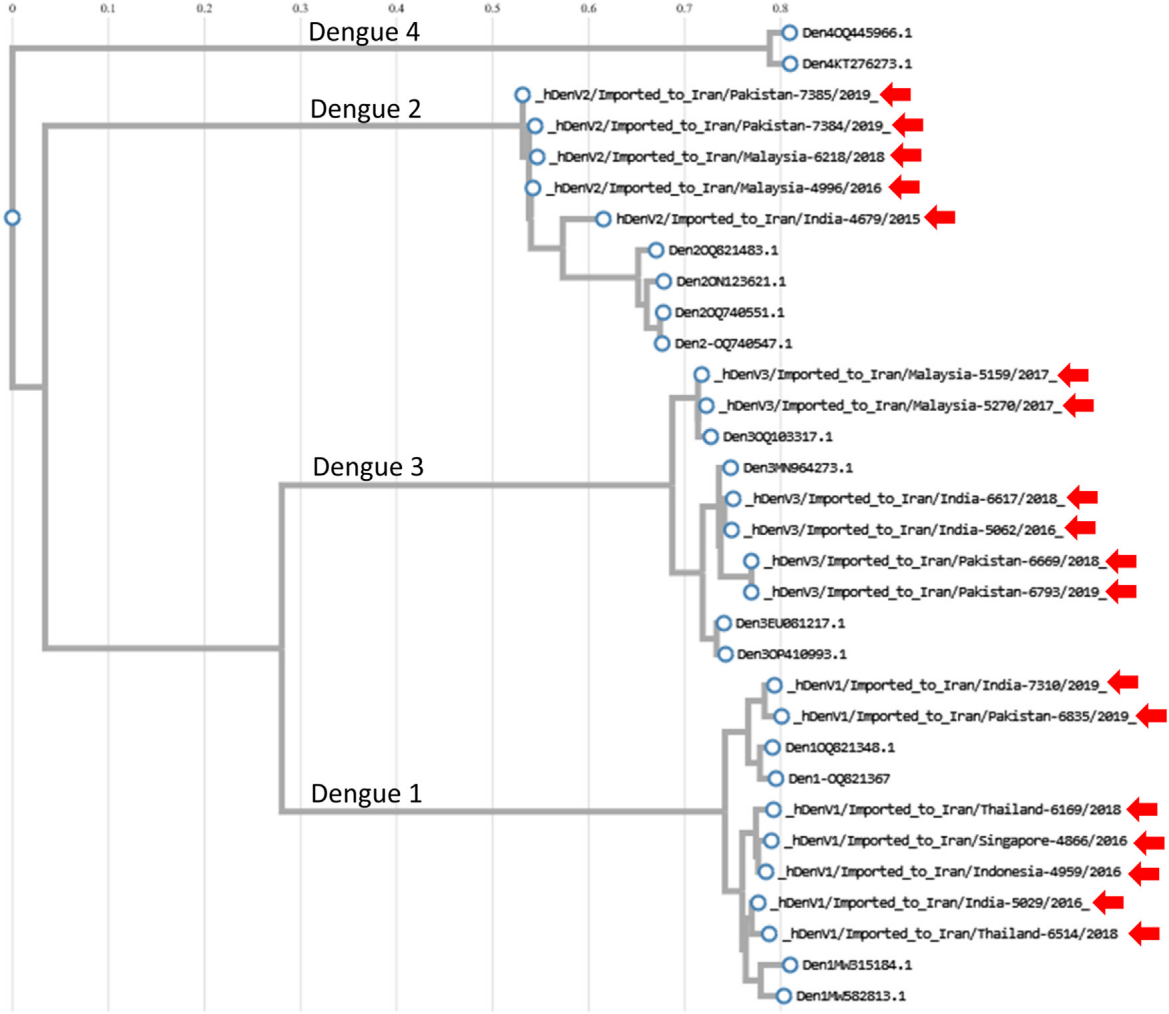
Phylogenetic tree of imported Dengue cases. The sequence alignment and phylogenetic reconstruction utilized the “build” function within ETE3, implemented on GenomeNet (https://www.genome.jp/). The dataset for this analysis combined sequenced samples from the current study with reference samples retrieved from GitHub. These reference samples boasted known genotypes and were prepared by the Grabo laboratory specifically for comparison purposes. FastTree version 2.1.8, employing default settings, constructed the phylogenetic tree. Sequences identified in this study are marked with red arrows for easy reference.

Figure 3 depicts a phylogenetic tree constructed to visualize the evolutionary relationships among imported dengue cases. The sequence alignment and phylogenetic reconstruction utilized the “build” function within ETE3, implemented on GenomeNet (https://www.genome.jp/). The dataset for this analysis combines sequenced samples from the current study with reference samples retrieved from GitHub. These reference samples contained known genotypes and were prepared by the Grabo Laboratory for comparison purposes. FastTree version 2.1.8, employing the default settings, was used to construct the phylogenetic tree. Sequences identified in this study are marked with red arrows for ease of reference.

**Figure 4:**
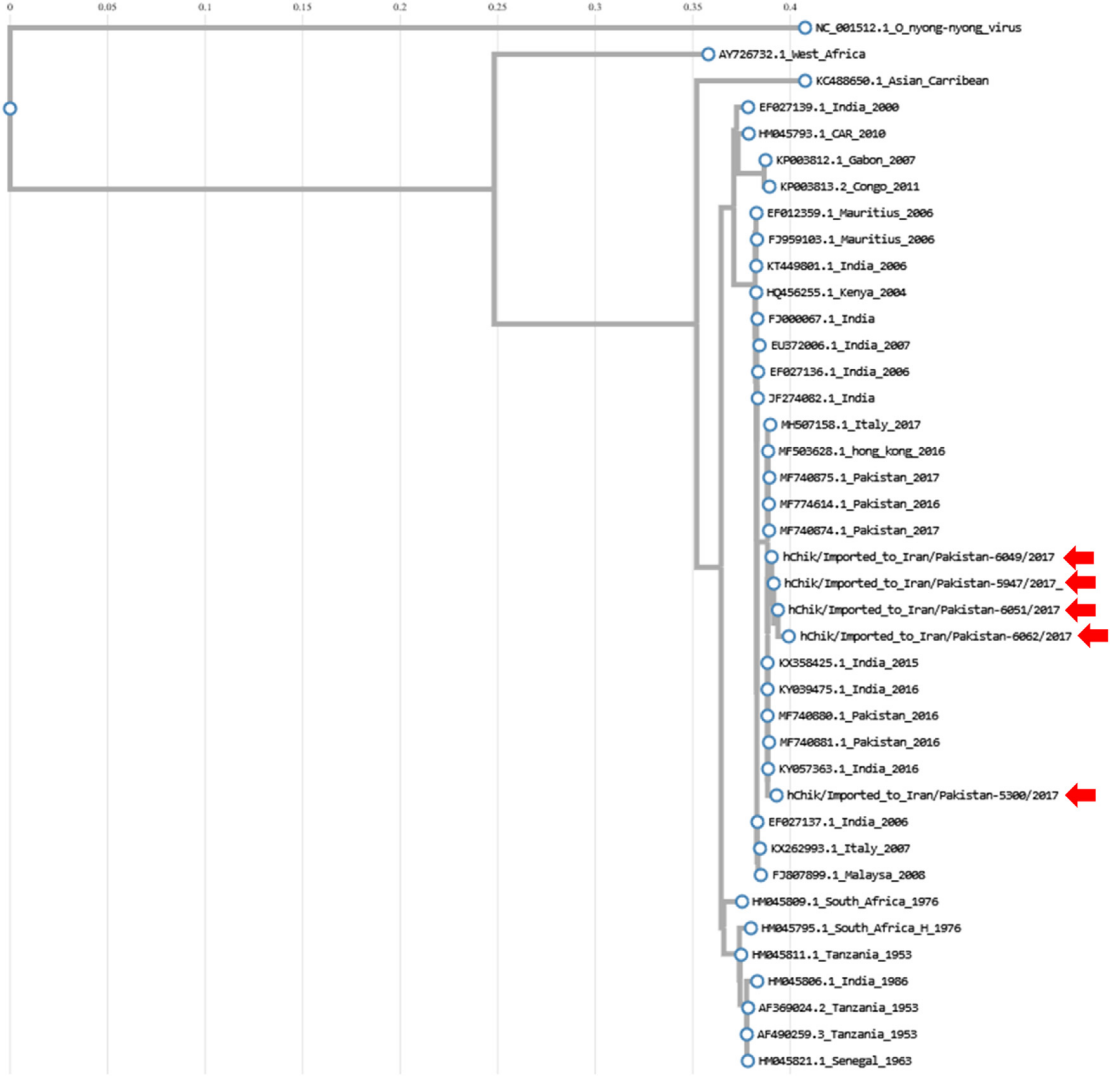
Phylogenetic tree of imported Chikungunya cases. The sequence alignment and phylogenetic reconstruction utilized the "build" function within ETE3, implemented on GenomeNet (https://www.genome.jp/). The dataset for this analysis combined sequenced samples from the current study with reference samples retrieved from GitHub. These reference samples boasted known genotypes and were prepared by the Grabo laboratory specifically for comparison purposes. FastTree version 2.1.8, employing default settings, constructed the phylogenetic tree. Sequences identified in this study are marked with red arrows for easy reference.

Unraveling the genetic makeup of chikungunya-positive cases revealed a captivating truth: all cases belonged to the Asian genotype (accession numbers: EPI_ISL_19388140, EPI_ISL_19388141, EPI_ISL_19388139, EPI_ISL_19388142, and EPI_ISL_19388138). This genotype shares a striking resemblance with the viral strains that caused the 2017 chikungunya outbreak in Pakistan. Figure 4 illustrates this relationship.

## Discussion

This study is a retrospective descriptive case series of dengue, chikungunya, and Zika viruses among febrile travelers to Iran between 2015 and 2021. The results of this study suggest that Iran's land border with Pakistan as the potential risk entry point for the introduction of both dengue and chikungunya into the country. Pakistan has a land border of 909 km with Iran. This border starts at the point of intersection of the borders of Iran, Pakistan, and Afghanistan and follows a relatively straight line towards the southeast of Iran until it ends in the Gulf of Oman. Due to the prevalence of dengue fever and chikungunya in India and Pakistan, as well as frequent border crossings by migrants, this region has been a risk area for imported dengue fever and chikungunya. As shown in this study, all imported cases of chikungunya disease from 2015 to 2021 were related to 2017 and coincided with an outbreak of this disease in Pakistan. Floods and climate change in Pakistan have led to outbreaks of dengue and chikungunya in 2016, 2017, and 2023 [11,12]. Considering the extensive borders of Iran and the cultural and kinship ties of the residents of the border areas of Iran and Pakistan, this land border has been a risk border for the import of diseases into the country in various years.

Cases imported through land transport constituted a significant proportion of all cases. This includes road transport on foot, by car, bus, trucks, and rail for commercial and familial visits among border residents, making frequent border crossings difficult to control. The second high-risk entry point is the air border, which introduces passengers from endemic countries, such as India and Malaysia. Air transport provides valuable information about passenger origins and destinations, aiding in tailored prevention and control strategies. For land and water borders, emphasis should be placed on the prevalence of dengue fever in neighboring countries, such as Pakistan and Iraq, and recently the Persian Gulf countries, such as Oman and the United Arab Emirates. Efforts should focus on increasing awareness and training residents in the border areas regarding dengue fever and chikungunya, encompassing infection prevention, disease symptoms, timely treatment, and enhancing monitoring systems. At air borders, priority should be given to monitoring passengers from high-risk areas. Implementing measures, such as body temperature checks, educational materials in airport waiting rooms, and informing travelers about disease prevention and control, can be effective.

From the viewpoint of sex, the number of imported cases from 2005 to 2021 was higher in men than in women. The reason for this phenomenon can be attributed to the difference in responsibilities and social and work activities between men and women, or they may have visited family or friends, and men may consult doctors more than women. In addition, men are more likely to travel abroad, both for business purposes and as migrant workers, which puts them at a higher risk of being bitten and infected with these diseases. Most imported cases were between ages 21 and 40 years. Considering that people in this group are of active working age, they are more likely to be exposed to mosquito bites and therefore, more likely to contract these diseases [13]. Therefore, it is logical to consider special training for this group and investigate them more seriously for viral infections upon their return from endemic countries.

Despite the increase in referrals to the reference laboratory since 2017, the number of referrals to the laboratory following the COVID-19 pandemic decreased sharply in 2020 and after the pandemic. Considering the cases of asymptomatic infections caused by these diseases, it is important that the surveillance system functions properly in searching for and referring to suspected and probable cases. Targeted training for doctors and health care workers and upgrading the laboratory diagnostic network are very effective for the accurate monitoring of these diseases.

The distribution of positive cases of the disease from 2015 to 2021, categorized by month, shows that in the rainy and hot months of the year, including June and October, suspected and positive cases increased, which is in line with the annual trend of mosquito-borne diseases in other parts of the world. This emphasizes the importance of intensifying the control of entry points and common borders of the country with endemic areas during these months of the year. Studies have shown that rainfall, temperature, and humidity are important climatic factors that affect mosquito density, and that weather conditions are favorable for mosquito breeding during these seasons [[14], [15], [16]].

This study emphasizes the importance of robust border surveillance systems in both endemic and non-endemic areas, including enhanced monitoring and health checks for travelers from endemic countries. In addition, regular training for health care professionals across various levels, from private clinics to central teaching hospitals, is crucial to improve their knowledge of dengue, chikungunya, and Zika. Strengthening the country's diagnostic capacity through network development and infrastructure upgrades is vital for early detection, which is critical for both disease control and treatment. Finally, fostering scientific and technical collaboration with neighboring endemic countries, particularly Pakistan, is essential for exchanging expertise, sharing experiences, and providing technical assistance in controlling these diseases.

By implementing these comprehensive measures, Iran can effectively prevent the further spread of dengue, chikungunya, and Zika, thereby minimizing their associated disease burden. Vigilant border surveillance, enhanced health care worker education, improved diagnostic capabilities, and international collaboration are fundamental for safeguarding public health.

## Conclusion

Our investigation of imported cases of dengue, chikungunya, and Zika viruses in Iran from 2015 to 2021 revealed a significant vulnerability along the country's land border, with Pakistan as a primary route for cross-border transmission, with the majority of imported cases occurring in males and individuals aged 21-40 years. Our investigation of imported cases of dengue, chikungunya, and Zika virus in Iran from 2015 to 2021 revealed significant vulnerability along the country's land border with Pakistan. This route emerged as the primary pathway multiple times during this period for the cross-border importation of these arboviral diseases. These findings highlight the critical importance of robust border surveillance in mitigating the introduction of vector-borne diseases.

This study underscores the need for a multifaceted approach to prevent and control the importation and potential establishment of these viruses in Iran. This strategy should prioritize the implementation of enhanced surveillance measures at land, air, and sea borders. Equally important are public education campaigns designed to raise awareness about the risks associated with these diseases as well as preventative measures and characteristic symptoms. Concurrently, it is essential that health care professionals are equipped with the necessary knowledge and skills for the accurate diagnosis, management, and reporting of imported cases. Strengthening laboratory diagnostic capacity is vital to ensure the timely and precise identification of infections. Furthermore, fostering international collaboration with neighboring countries, particularly those with established endemicity, is crucial for sharing information, best practices, and resources for effective disease control.

By adopting this comprehensive strategy, Iran can effectively safeguard its population from increasing threats posed by these emerging infectious diseases.

## Declarations of competing interest

The authors declare that they have no known competing financial interests or personal relationships that could have appeared to influence the work reported in this paper.

## Appendix Supplementary materials

Image, application 1

## Footnotes

Supplementary material associated with this article can be found, in the online version, at doi:10.1016/j.ijregi.2025.100593.
