# Supplementary material for: Dengue and chikungunya viruses among febrile travelers entering Iran (2015-2021): Evidence of multiple introductions from different countries
Source: IJID Reg. 2025 Feb 1;14:100593. doi: 10.1016/j.ijregi.2025.100593 (PMC11919373; doi:10.1016/j.ijregi.2025.100593)
Supplement: Supplementary file 1 [file mmc1.docx]

# Supplementary Data:

S1

| Table 1 – Primers and Probes for Multiplex Real-Time RT-PCR | | | |
| --- | --- | --- | --- |
| Ref | Sequence | Primer Name | Virus |
| ^[5]^ | GGCAAACGCAGTGGTACTTCCT | Chik As | Chikungunya |
|  | TGATCCCGACTCAACCATCCT | Chik S |  |
|  | TCCGACATCATCCTCCTTGCTGGC | Chik P |  |
| ^[6]^ | GGATAGACCAGAGATCCTGCTGT | Den S | Dengue |
|  | CATTCCATTTTCTGGCGTTC | Den As |  |
|  | CAATCCATCTTGCGGCGCTC | Den AsPlus |  |
|  | CAGCATCATTCCAGGCACAG | Den p |  |
| ^[7]^ | TTGGTCATGATACTGCTGATTGC | Zik S1 | Zika |
|  | CCTTCCACAAAGTCCCTATTGC | Zik As1 |  |
|  | CGGCATACAGCATCAGGTGCATAGGAG | Zik P1 |  |
|  | CCGCTGCCCAACACAAG | Zik S2 |  |
|  | CCACTAACGTTCTTTTGCAGACAT | Zik As2 |  |
|  | AGCCTACCTTGACAAGCAGTCAGACACTCAA | Zik P2 |  |
| ^[17]^ | AGATTTGGACCTGCGAGCG | RNase P F | IC (RNase P) |
|  | GAGCGGCTGTCTCCACAAGT | RNase P R |  |
|  | TTCTGACCTGAAGGCTCTGCGCG | RNase P Probe |  |

S2

| Table 2 – Sequencing Primers | | | |
| --- | --- | --- | --- |
| Ref | Sequence | Primer Name | Virus |
| [8] | ACAAAACCGTCATCCCGTCTC | Chik E1 F | Chikungunya |
|  | TGACTATGTGGTCCTTCGGAGG | Chik E1 R |  |
| [9] | TCAATATGCTGAAACGCGCGAGAAACCG | Den 1 | Dengue |
|  | TTGCACCAACAGTCAATGTCTTCAGGTTC | Den 2 |  |
| [10] | [10]AATGTACGCTGATGACACAGCTGGCTGGGACAC | Zika F | Zika |
|  | TCCAGACCTTCAGCATGTCTTCTGTTGTCATCCA | Zika R |  |

S3


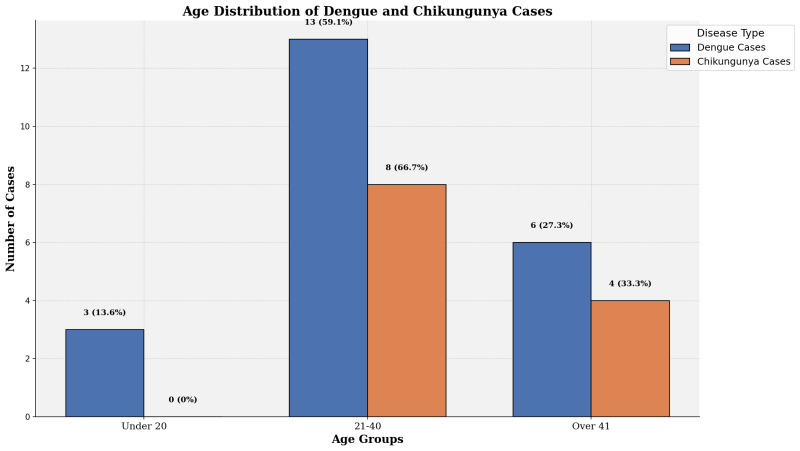


Fig1- Age distribution of positive cases of imported Dengue and Chikungunya 2015-2021
